# Comparison of Hands-On Versus Online Learning in Teaching Ultrasound Skills for Achilles Tendon Rupture: A Pilot Study

**DOI:** 10.7759/cureus.9021

**Published:** 2020-07-06

**Authors:** Devjani Das, Monica Kapoor, Cara Brown, Abbas Husain, Marina Rubin, Jerel Chacko, Simone Rudnin, Barry Hahn, Josh Greenstein

**Affiliations:** 1 Emergency Medicine, New York Columbia Presbyterian Hospital, New York, USA; 2 Emergency Medicine, UMass Memorial Medical Center, Worcester, USA; 3 Emergency Medicine, The Mount Sinai Hospital, New York, USA; 4 Emergency Medicine, Staten Island University Hospital, Staten Island, USA; 5 Emergency Medicine, Newark Beth Israel Medical Center, Newark, USA

**Keywords:** achilles, rupture, ultrasound

## Abstract

Introduction

In the emergency department, the diagnosis of an Achilles tendon rupture (ATR) is reportedly missed in greater than 20% of cases. A limited number of studies evaluate the use of cadaver models as a potential ultrasound teaching and training modality. We hypothesize that emergency medicine residents can effectively utilize point-of-care ultrasound (POCUS) on cadaver models and a focused teaching intervention to assess their ability to detect ATRs.

Methods

A prospective study of 23 EM residents was performed. All participants in the study were divided into two learner groups: (a) independent and (b) hands-on. The independent learner group received a 30-minute online didactic lecture demonstrating how to diagnose ATRs. The hands-on learner group received direct instruction on cadaver lower leg models with a ruptured and normal Achilles tendon (AT). Both groups then participated in identifying either normal or ruptured ATs on six cadaver lower leg models.

Results

The sensitivity and specificity were 89% and 82% in the independent learner group 96% and 100% in the hands-on learner group, respectively. The overall sensitivity and specificity were 91% and 88%, respectively. There was a trend toward successful identification with increased years of residency training.

Conclusions

In this study, lower leg and ankle cadaver models were found to be as effective as an independent learner model for potential POCUS teaching and training modality in both novice and more advanced trainees.

## Introduction

The Achilles tendon (AT) is considered the strongest tendon in the body. However, it is also the most commonly injured tendon in the ankle [1]. AT ruptures (ATRs) are relatively common, occurring in 18 per 100,000 persons. ATRs are often a result of overuse from activities involving chronic repetitive tensile forces, medication use, or comorbid conditions. More than 80% of all ruptures occur between two and six centimeters above the calcaneus [2]. In the emergency department (ED), an AT injury diagnosis is often made solely on clinical examination. Findings may include increased passive ankle dorsiflexion, weak plantarflexion strength, and a palpable defect over the tear. Physical examination alone has been reported to miss greater than 20% of cases of acute ATRs. This finding may be due to the pain and swelling associated with the injury, as well as the difficulty in identifying incomplete ATRs. Missing an ATR can result in delayed diagnosis and unnecessary morbidity [1-4].

Ultrasound (US), when performed by a radiologist, is reliable in diagnosing an ATR. Prior studies have found that US sensitivity and specificity for complete and incomplete ATRs are 96-100% and 83-100%, respectively [1,5]. The advantages of US include its wide availability, portability, safety, and favorable time and cost factors. Several case studies have shown point-of-care US (POCUS) to be useful in the acute setting for diagnosing an ATR [6]. There are few studies evaluating the use of cadaver models for POCUS education. However, results suggest that cadavers are an effective teaching model [7,8]. We hypothesize that emergency medicine (EM) residents can effectively utilize POCUS on cadaver models along with a focused teaching intervention to assess their ability to detect ATRs.

## Materials and methods

This was a prospective cohort study of EM residents at Staten Island University Hospital (SIUH). The study was conducted during a single educational session in January 2017 at an affiliated teaching site of the study hospital. SIUH is a 700-bed tertiary-care urban teaching hospital. The hospital maintains a three-year ACGME (Accreditation Council for Graduate Medical Education) accredited EM residency program consisting of eight residents per year. The study was granted an exemption by the local Institutional Review Board.

Study participation was optional and did not interfere with the educational session. Twenty-three residents were consented and enrolled for the study. One resident did not participate in the educational portion of the session and was not included. Study participants were assigned to one of two teaching intervention groups. Before the teaching interventions, study participants were given a brief six-question online questionnaire. This questionnaire included questions regarding each resident’s experience and comfort level using POCUS for musculoskeletal applications. Study participants were then assigned to one of two teaching groups.

The first study group (group A) was an independent learner group. Each participant in this group received a 30-minute computerized didactic lecture, which was created by the study investigators before the educational session (Video 1).

**Video 1:** Computerized didactic lecture on how to utilize ultrasound to diagnose Achilles Tendon pathology

The lecture depicted how to utilize POCUS to diagnose an ATR and included images of normal and abnormal AT pathology. Also included was which transducer to use for the study, how to optimize the US image to best visualize the AT, and how a normal and an abnormal AT appeared.

The second study group (group B) was a hands-on learner group. Each participant in this group received a 30-minute hands-on teaching session with a fellowship-trained US faculty member. During this session, the participants examined normal and abnormal AT pathology on two separate cadaver models. The US faculty member demonstrated which transducer to use for the study, how to optimize the US image to best visualize the AT, and how a normal and an abnormal AT appeared. The participants in group B were then allowed to use POCUS on the cadaver models to practice their skills. The lower leg and ankle models used for the training session were not re-used in the study.

**Figure 1:**
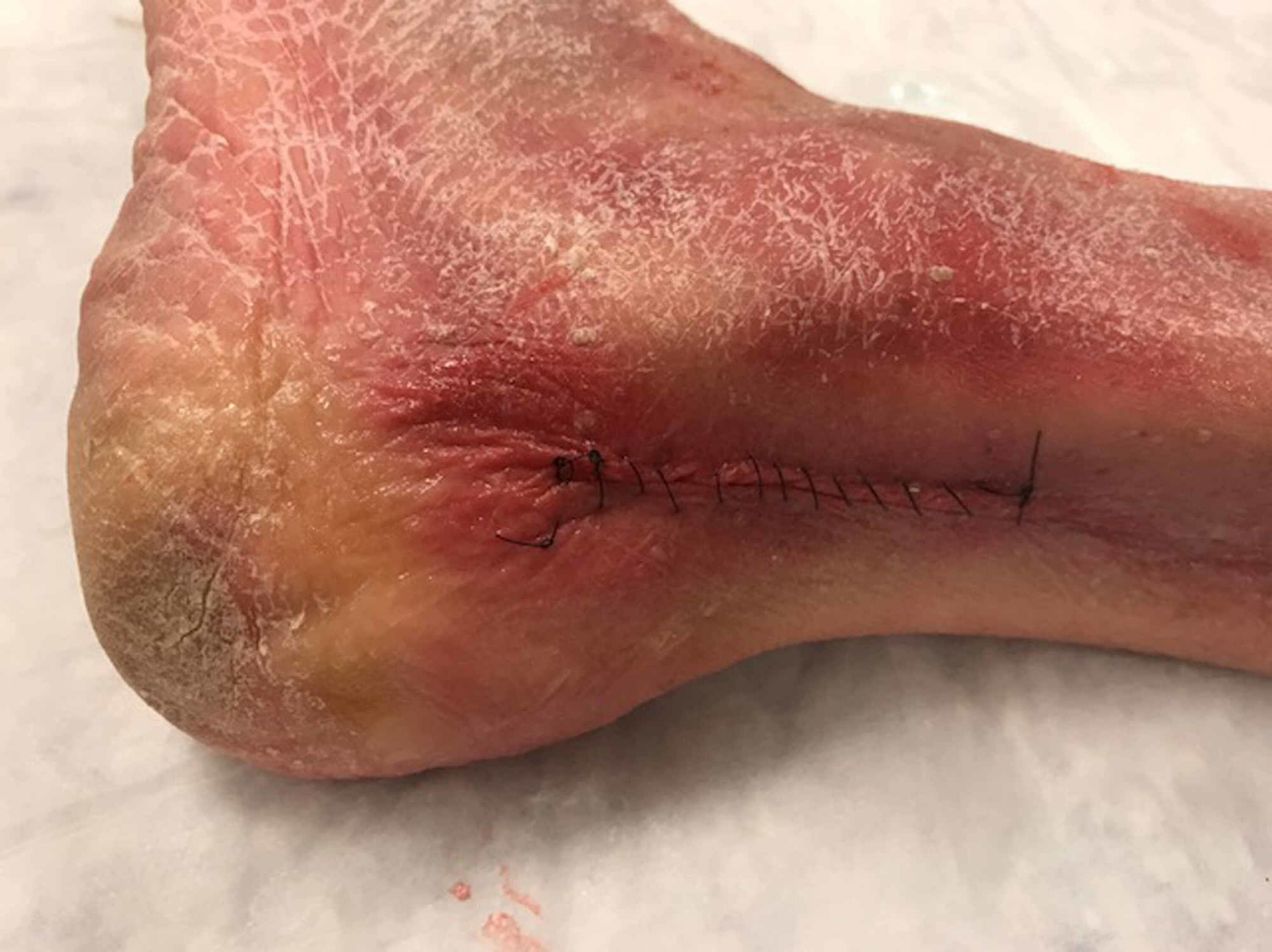
Single cadaver lower leg and ankle models used with sutures over the area of incision and possible Achilles tendon rupture.

After each group completed their learning modules, both groups participated in identifying AT pathology on six cadaver lower leg and ankle models. Just before the educational session, investigators created a vertical incision in the mid-calf region of all six cadaver lower leg and ankle models. Three models had an ATR simulated by study investigators by using a scalpel to lacerate the AT. The incisions of all six models were subsequently sutured closed (Figure 1).

**Figure 2:**
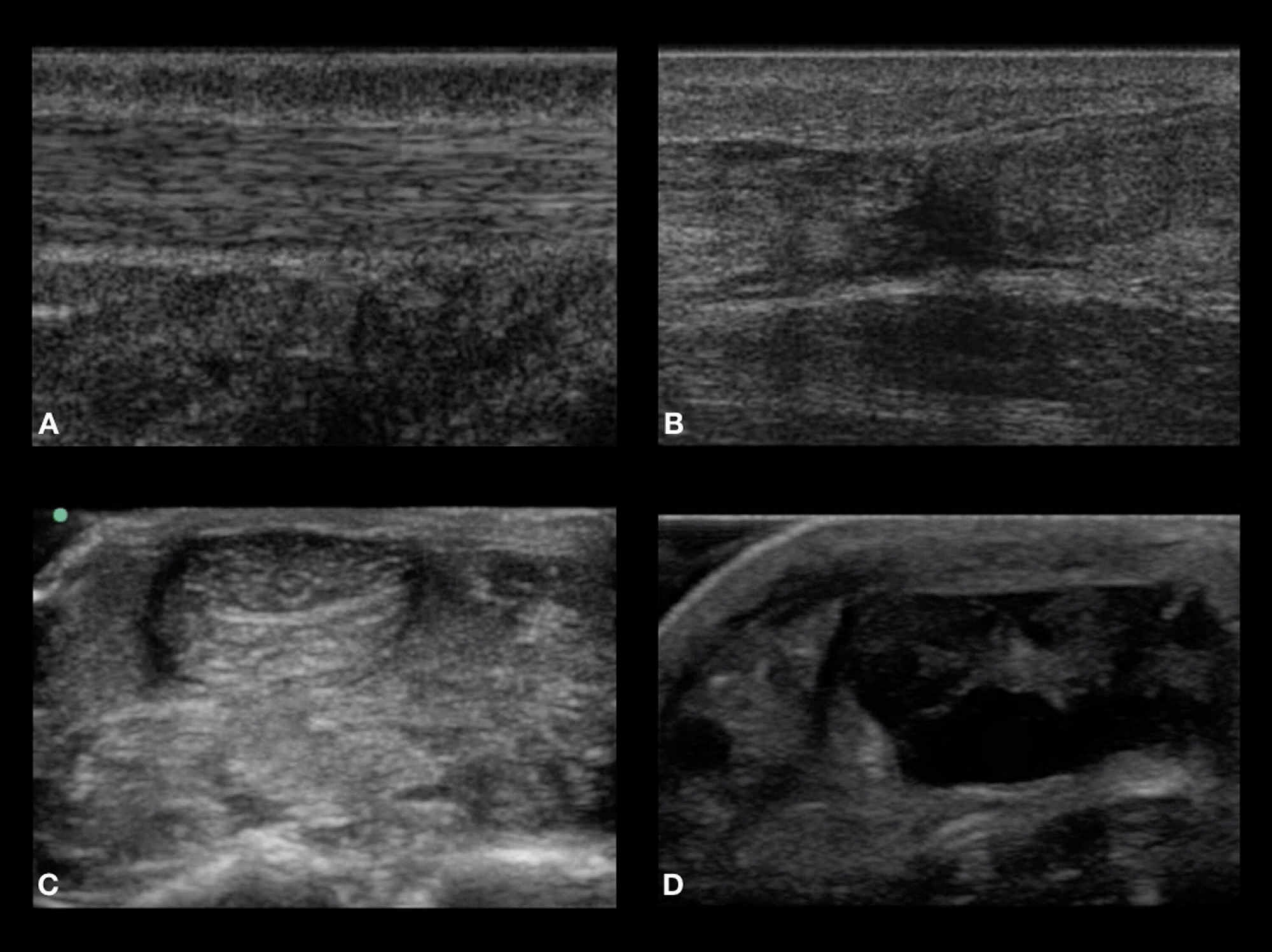
Panels A and C represent the normal sonographic Achilles tendon appearance in the sagittal and transverse planes, respectively. Panels B and D represent ruptured Achilles tendons seen in the sagittal and transverse planes, respectively. All images obtained were from actual cadavers used.

Each model was placed on an individual table with a US machine adjacent to it. All participants used a high-frequency linear transducer to perform the examination. The six specimen tables were strategically placed so that only one resident was present at each table. Residents were instructed not to discuss the results of each model. Participants were given up to two minutes per model to use POCUS to diagnose a normal or ruptured AT on each of the six models (Figure 2).

Participants were instructed to fill out a paper form indicating whether each model was positive for an ATR, negative for an ATR, or if the AT was not visualized.

The data were stored using Research Electronic Data Capture (REDCap), a secure web-based application designed to support data capture for research studies. The data were analyzed using descriptive statistical methods and were expressed as frequency counts and percentages for categorical variables. Sensitivity and specificity were calculated for the POCUS test, with subgroup analyses by the learner group. Results were presented with 95% confidence intervals. Data analyses were conducted using Analyse-it Version 5.11.1 (Analyse-it Software, Ltd., Leeds, UK). Significance was defined as a p-value of less than 0.05.

## Results

All 23 EM residents participating in the educational session were enrolled and included in the final analysis. Eight were assigned to the hands-on learner group (three post-graduate year [PGY] 1, three PGY2, and two PGY3 residents) and 15 to the independent learner group (five PGY1, five PGY2, and five PGY3 residents). All PGY2 and PGY3 residents had completed US rotations. Only five (63%) of PGY1 residents had completed US rotations before this investigation. Despite this, only six (86%) of PGY3, five (63%) of PGY2, and no PGY1 residents had previously received instruction on the diagnosis of ATR using POCUS. Of the 11 (73%) participants who did previously learn how to diagnose an ATR using POCUS, only four (27%) had previously attempted to identify an ATR using POCUS. Residents reported increasing comfort level of performing and interpreting ATR on US, with increasing years of residency. This was consistent with the trend toward greater comfort performing and interpreting musculoskeletal US with increased years of residency training.

The overall sensitivity and specificity were 91% (95% CI: 82-96) and 88% (95% CI: 79-94) for diagnosing the presence of an ATR. The negative predictive value for this group was 96%. The sensitivity and specificity were 96% (95% CI: 80-99) and 100% (95% CI: 86-100) in the hands-on learner group and 89% (95% CI: 77-95) and 82% (95% CI: 69-91) in the independent learner group, respectively (Table 1).

**Table 1:** Sensitivity and specificity in identifying normal or ruptured Achilles tendon by teaching intervention group, year of training, and overall.

	Sensitivity	Specificity	Negative Predictive Value	Positive Predictive Value
Overall	91%	88%	91%	91%
Independent group	89%	82%	88%	83%
Hands-on group	96%	100%	96%	100%
Post-graduate year 1	83%	75%	77%	82%
Post-graduate year 2	96%	96%	96%	96%
Post-graduate year 3	95%	95%	95%	95%

The NPV for this group was 88%. As shown in Table 1, there was a non-statistically significant trend toward successful identification with increased years of residency training.

## Discussion

Human cadaver models have been effectively used to teach US diagnosis of pathological conditions, such as abscesses and pneumothoraces [8-11]. Our study used human cadaver lower leg and ankle models to teach POCUS diagnosis of ATRs. We were able to demonstrate that residents who had never, or rarely, used POCUS to diagnose an ATR were comfortably able to do so after either a minimal hands-on or independent computer-based learning.

Recent advances in simulation-based teaching have shifted residents’ training toward using simulation models for hands-on learning for a multitude of medical interventions. Studies have shown that simulation-based teaching may improve patient safety through specific training and protocols [10]. According to recent literature, learners feel that using cadavers provides them with a more practical simulation resource [12]. The act of visualizing and practicing hands-on techniques on cadavers has been advocated as a worthwhile educational tool [13,14].

Prior studies have shown that more than 20% of all cases of ATRs are initially misdiagnosed [15]. Reasons for the missed diagnosis in the ED include, but are not limited to, the rarity of the diagnosis, subtle histories, and unclear physical examination findings [16]. If the appropriate treatment is delayed, patients will almost certainly require surgical intervention [15].

Clinicians who suspect an ATR may perform several clinical tests. This includes the well-known Thompson’s test in which the patient lies prone with the injured foot hanging over the end of a table, and the clinician squeezes the calf distal to its maximal girth in order to stimulate plantar flexion. The absence of this flexion is a reliable indicator of an ATR [17]. However, this test, in addition to a few other clinical examinations, though useful, may be challenging to perform in the acute setting when patients present in pain. It is useful to have adjunctive tests to aid in diagnosing an ATR in the acute setting. Radiography may be used, but the radiographic findings are subtle, widely missed by many clinicians, and have mostly fallen out of favor. MRI may also be utilized but is expensive, time-consuming, inferior to US, and often not readily available or necessary to make the initial diagnosis of an ATR [15,16,18].

In the acute setting, POCUS is useful for diagnosing an ATR, with prior studies showing pooled sensitivity and specificity of 96-100% and 83-100%, respectively [5,17]. Our study demonstrated similar findings. Prior studies have also shown that US may be useful in reliably estimating the severity of an injury and determining whether surgical intervention may be required [17,19-21]. US not only allows the visualization of the complete disruption of the normal parallel pattern of fibers within the tendon but also allows for additional findings of a hematoma formation, herniation of Kager’s fat into the tendon gap, and visualization of the plantaris tendon. In addition, the entire examination may be performed with dynamic sonography as the patient can dorsi- and plantarflex on command, which may further aid in diagnosis [20].

The limitation of using POCUS for the diagnosis of an ATR, as with any application of US, is operator dependence [17,22]. To reduce diagnostic errors in US acquisition and image interpretation in POCUS, clinicians need to become familiar with differences in physiological and pathological processes and understand when an image is inadequately optimized [17,22]. In this study, it was found that with minimal training, whether by independent learning module or hands-on learning with cadaver models, residents were able to identify an ATR successfully. The trend toward successfully identifying an ATR with increased years of residency training is likely related to the residents’ prior use of POCUS for musculoskeletal applications previously during their residency training.

There were several limitations to this study. This was a single-center study with a limited number of participating residents, limiting the generalizability of the results. Another limitation was that the ATRs simulated on the cadaver models were incisions made to the AT with scalpels rather than a natural rupture, as encountered with patients in the ED. In addition, the cadaveric calf muscles and AT did not shorten after the incision was made using the scalpel, as it usually would in an acute setting. Also, in the acute setting, hematomas are often useful adjuncts to finding the location of an ATR [18]. Prior to data acquisition, attempts were made to simulate hematoma formation around the artificially created ATRs. However, the study investigators did not find any of the simulated hematoma formations to be adequately representative of an acute hematoma and, therefore, were not used during the data acquisition portion of the study.

Prior studies have also confirmed that US, although helpful, may not be sufficiently reliable for the diagnosis of partial ruptures of the AT [22]. As this was an initial teaching session for resident ultrasonographers with varying experience using US, the study investigators chose not to incorporate partial tears with complete tendon tears among its cadaver models to confound the results of the study. One of the most significant advantages of using POCUS in real time is that a patient can indicate where his or her pain is located and comply with dynamic US imaging by plantar- or dorsiflexing the calf. Without these helpful adjuncts to the US examination, it may be more challenging to make the ultimate correct diagnosis of an ATR. In addition, all cadavers used for the study were fresh frozen cadavers with no preservatives used. Each cadaver had its own unique set of properties with a variable amount of fluid and fat retention, which affected the quality of US images obtained, with some cadavers providing greater image resolution than others. Furthermore, cadavers are not always readily available. This affects both the ability to potentially utilize cadavers as a teaching modality as well as the ability to adequately obtain a sufficient sample size to study the effectiveness as a teaching tool. Lastly, this study is unable to assess whether this skillset in ATR sonography will persist longitudinally, a problem endemic to may less common US modalities.

## Conclusions

Misdiagnosing an ATR in the ED may result in profound patient morbidity. Although an ATR is a clinical diagnosis, additional diagnostic tools may be useful. US has shown to be one such diagnostic tool.

In this study, lower leg and ankle cadaver models were found to be as effective as an independent learner model for potential POCUS teaching and training modality in both novice and more advanced trainees. Residents with limited POCUS experience were highly successful in identifying a normal versus ruptured AT after a brief training session using cadaver models.

## Figures and Tables

**Figure 3:**
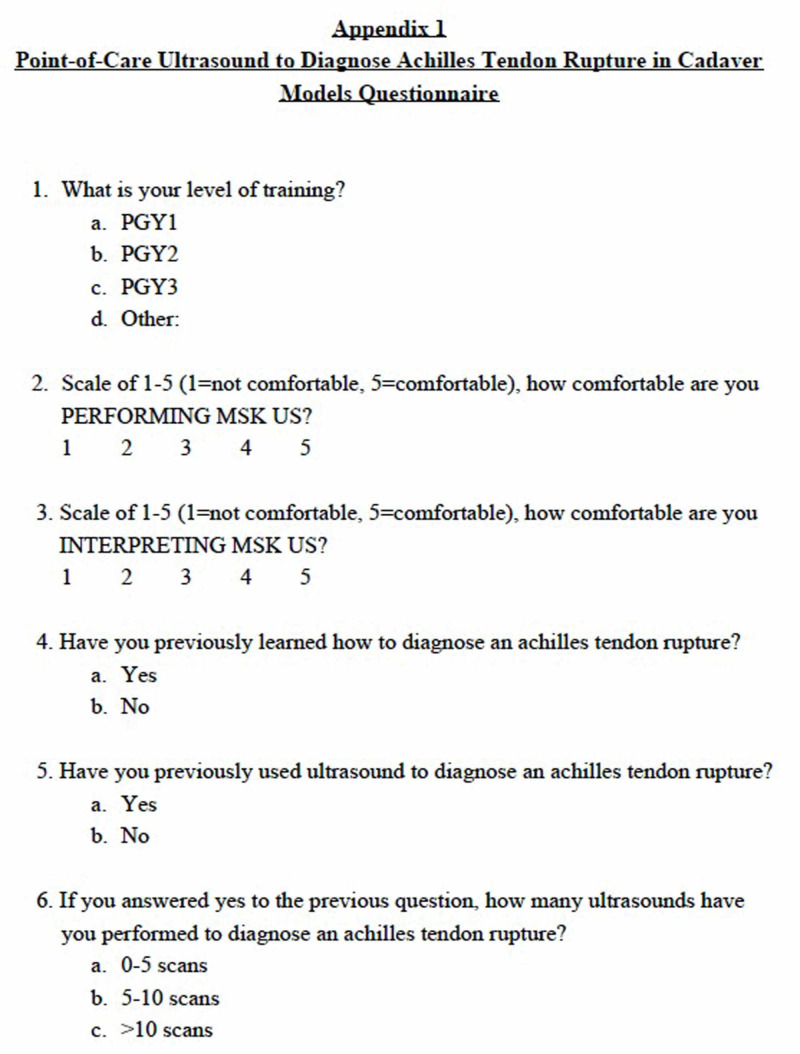
Six-question online questionnaire. PGY, post-graduate year; MSK, musculoskeletal; US, ultrasound
